# The Gospel of Self-Help

**Published:** 1918-07-20

**Authors:** 


### A TRUMPET-CALL TO NURSES.

### The Gospel of Self-Help.

It may presumably be taken as proven that the economic condition of the nursing profession is lower than that of any corresponding class of workers, and that this condition applies even to hospitals like "the London." I am in no respect concerned with the good form or the propriety of Major Chapple's Parliamentary performance chonicled in last week's Hospital, but I must protest that Lord Knutsford's disclaimer in the *Times*, based on the fact that "the London" nurses prefer to remain in the hospital's service to venturing forth "on their own," fails to convince. We all know that this is a free country, and that Britons never, never shall be slaves. But it does not, therefore, follow that their conditions of employment are just, and that their occupations are those which they prefer. This by the way. It is extremely improbable that anybody, including Lord Knutsford, will maintain that British hospital nurses generally are adequately remunerated. I venture, indeed, to prophesy that one of the first hospitals to proclaim betterment will be his own.

### Why are Some Nurses Overworked and Underfed?

If my premises are granted I desire to point out that the great majority of those most concerned do not understand the underlying cause, and the importance of its being made clear is obvious. Even the junior ward sister realises how hard it is to provide an effectual cure for any disease whose origin is obscure. The physician may look for symptoms, and treat them as best he may, but until the germ or microbe or whatever may be primarily responsible is laid bare by science and research, he is comparatively helpless. Why is the nurse overworked and underpaid? To this question it is of the utmost importance that a convincing answer should be given. One operating factor may be that the nurse is usually the servant of a necessitous charitable institution; but this is by no means the sole cause, for, if so, all other hospital workers would be in similar plight. And they are not.

### Why Don't the Nurses Help Themselves?

I wonder if it has ever occurred to the nurse that *she takes absolutely no active part in endeavouring to help herself.* This may seem a striking statement, but I ask her to look around and ascertain if it is true. Despite the volume of hypocritical nonsense that has been written and preached about "laymen" interfering in the nurse's affairs, it must be admitted that she has no champion within the fold. All her friends are outsiders. Look around and pick out the people whose names have become prominent. Begin with Queen Alexandra: then take such women as the Marchioness of Dufferin and the Countess of Dudley. Include Mrs. Bedford Fenwick, if you have a mind. Her occupation is that of a journalist—a layman abusing laymen. Then you have Sir Arthur Stanley heroically endeavouring to stir up the nation to a sense of its indebtedness, Major Chapple leading his weather-beaten battalion of Registrationists, Lord Knutsford, and all the other names that may occur to the reader. I am not here and now concerned with appraising or comparing the merits of those whom I have mentioned. I merely desire to show that the prominent champions are of the lay element rather than women earning their livelihood as nurses—this, and nothing more. It may possibly be said in reply that naught else is possible, which I neither affirm nor deny. I am content for the present with chronicling what I believe to be true, and in asking my readers to verify what I say. The working nurse not only does nothing to help herself, but in addition she is inarticulate. The only evidence of voice symptoms that I have heard is Lord Knutsford's, to the effect that there is a growing tendency to growl. It is a good word. My exceptionally intelligent dog cannot quite speak, but he is able to express himself wonderfully well otherwise. It would, therefore, appear that the highest form of expression to which British nurses have attained is, in actual fact, growling, and even this is rare; speaking generally, the profession is, with one accord, dumb.

### Thirty Lean if not Barren Years.

These may be hard and unpleasant words; but they will out. They are, however, uttered with good motive, and remember we have been in search of a microbe. If we have discovered it, we can proceed to seek a remedy that will effect a cure rather than go on tinkering as we have been doing for thirty years. This, I believe, according to the apostles, covers the Registration agitation era, and by common consent these years have been lean if not barren. *If the nurse will progress she must help herself.* Hear my old friend Samuel Smiles on this subject: "The spirit of self-help," he observes, "is the root of all genuine growth in the individual; and exhibited in the lives of many it constitutes the true source of national vigour and strength. Help from without is often enfeebling in its effects, but help from within invariably invigorates. Whatever is done for men or classes, to a certain extent takes away the stimulus and necessity of doing for themselves." And again: "Even the best institutions can give a man no active help. Perhaps the most they can do is to leave him free to develop himself and improve his individual condition. But in all times men have been prone to believe that their happiness and well-being were to be secured by means of institutions rather than by their own conduct. Hence the value of legislation as an agent in human advancement has usually been much over-estimated." Substitute "nurses" for "men" in these passages and consider their application.

### The Imperative Need of Unity.

To come to practical politics. I shall be asked, What can a body of hard-worked women do, as individuals or collectively? To this I have a simple answer, so simple that, as in the case of the leprous warrior, I fear it may be scorned. "If the prophet had bid thee do some great thing, wouldest thou not have done it?" *The first elementary, obvious, and necessary thing to do is to unite.* I recently heard a somewhat cynical statesman remark—"Women never did and never will unite." As a historian he is right: as a prophet he may be right. I do not know. But of this I am convinced, that without union there can be no progress. A bark here, a growl there, a screech or a speech by somebody else outside the pale—all these are ineffectual. For the first time in the history of British nursing there has been and is established a machine that may become all-powerful; but without motive power it is useless. The wheels and cogs and engines may be of perfect workmanship, but the fires must be lighted. And the question is, Will nurses do their bit? Sir Arthur Stanley has sounded the trumpet on a hundred platforms, but how many have responded? One in seven, I am informed. It is a poor show. I can understand one in seven failing to respond, and I ask the six who stand and wait how can they expect a nation to become aroused while they themselves display such apathy?

### The Golden Hour for Nurses.

The College of Nursing is an institution that our grandmothers would have regarded as an impossible ideal, just as a few years ago we regarded flying. The College has arrived: it is in our midst. It stands for union and for progress. Its mission is, to voice the inarticulate nurse. The nurse may be fettered in many ways, but she can help by adding one more to the union, and when she joins she can become a recruiting agent and a missionary in the cause. She can wander into the highways and byways and compel them to come in. Even the irreconcilable may be persuaded to join the parade. The hour is a golden one. If it is neglected, when the war is over the nurse will be forgotten, and, like many a gallant Crimean veteran, may end her days in the workhouse.

"If the prophet had bid thee do some great thing.." !

IERNE.

